# Acute toxicity profile and treatment response of image guided hybrid brachytherapy using Venezia applicators in locally advanced cervical cancer: a single-centre experience in Pakistan

**DOI:** 10.3332/ecancer.2025.1883

**Published:** 2025-04-01

**Authors:** Javeria Haider, Humera Mahmood, Muhammad Faheem

**Affiliations:** Department of Oncology, Atomic Energy Cancer Hospital NORI, Islamabad 44000, Pakistan

**Keywords:** cervical cancer, concurrent chemoradiotherapy, hybrid brachytherapy, human papilloma virus

## Abstract

**Objective:**

To evaluate the efficacy and acute toxicity of hybrid brachytherapy using Venezia applicators in patients with locally advanced squamous cell carcinoma of the cervix.

**Methods:**

This prospective study involved 41 patients treated with external beam radiotherapy (EBRT) followed by brachytherapy. Patients received EBRT doses of 45–50.4 Gy with or without simultaneous integrated boost and concurrent chemotherapy. Brachytherapy was administered using Venezia applicators, delivering high-risk clinical target volume (HRCTV) doses of 80–90 Gy or >90 Gy. Treatment responses and toxicities were assessed using Response Evaluation Criteria in Solid Tumours Criteria 1.1, Common Terminology Criteria for Adverse Events Version 5.0, respectively.

**Results:**

After five visits, 65.9% of patients achieved complete response, 29.3% partial response and 4.9% stable disease. Acute toxicities were primarily Grade 0–1, with no Grade III or IV toxicities observed. Complete responders exhibited higher rates of Grade 0 toxicities across various parameters, including urination frequency and abdominal pain. Middle-class patients showed higher response rates, although this was not statistically significant. 46.3% Human Papillomavirus positive patients converted to negative status after treatment. There was no significant correlation of response rate with disease stage, EBRT dosage or duration of treatment.

**Discussion:**

Hybrid brachytherapy using Venezia applicators allowed for high-dose delivery to HRCTV without exceeding organ tolerance limits, resulting in effective local control (LC) and minimal acute toxicities. The study underscores the potential of hybrid brachytherapy in improving outcomes for cervical cancer patients, particularly in low-middle-income countries. Challenges included small sample size and patient follow-up limitations.

**Conclusion:**

Hybrid brachytherapy with Venezia applicators is effective and safe for locally advanced cervical cancer, providing high LC with minimal acute toxicity.

## Introduction

Cervical cancer is the fourth most frequently diagnosed cancer and the fourth leading cause of cancer death among women, with an estimated 604,000 new cases and 342,000 deaths worldwide in 2020 [1]. It is the third most common malignancy among women in Pakistan [2] which ranks seventh globally in cervical cancer mortality [3]. According to the Pakistan Atomic Energy Commission cancer registry, cervical cancer is the fourth most common cancer among women in the country [4]. There were 110 cases of cervical cancer out of the 7,000 new cases registered at the Oncology Department of NORI from June 2022 to January 2024.

The higher prevalence of cervical cancer in developing countries is attributed to factors such as inadequate Human Papillomavirus (HPV) screening, low socioeconomic status and poor genital hygiene [5]. The human development index and poverty rates account for more than 52% of the global variance in mortality [1].

Standard treatment for locally advanced cervical cancer involves external beam radiotherapy with concurrent weekly platinum-based chemotherapy, followed by brachytherapy, typically completed within 7–8 weeks [6]. Compared to traditional X-ray-based brachytherapy, 3-D planning using computed tomography (CT) and magnetic resonance imaging (MRI) offers a more precise volume-based dose calculation for the tumour and adjacent organs at risk (OARs), such as the bladder, rectum and sigmoid. This allows higher doses to be delivered to the tumour without exceeding OAR tolerance limits [3].

Standardised guidelines for 3D image-guided brachytherapy (IGBT) have established a uniform international approach to treatment and reporting clinical outcomes, leading to improved survival rates and reduced toxicity [6, 7] .

Radiotherapy can cause acute radiation toxicity (ART) within 90 days of treatment initiation and/or chronic radiation toxicity months or years after treatment completion. Over 80% of patients experience some form of ART during radiotherapy for cervical cancer, with the most common being haematological, gastrointestinal (GI) or genitourinary (GU) toxicity [8, 9]. The severity of these adverse effects depends on factors such as radiation dosage, technique, fractionation regimen, treatment duration and patient characteristics, including age, disease stage and comorbidities [8].

Radiation-induced toxicity, particularly acute GI, GU, haematological and skin toxicity like cytopenia, vomiting and diarrhea, can necessitate extensive intervention, increasing the cost of long-term treatment. Thus, identifying and monitoring patients at risk of ART is crucial.

Advancements in brachytherapy, particularly IGBT, have enabled excellent tumour control without exceeding OAR tolerance limits. In this study, patients with locally advanced squamous cell carcinoma (CA) of the cervix were treated with external beam radiotherapy (EBRT) using intensity-modulated radiotherapy (IMRT) at a total dose of 45 Gy with 1.8 Gy per fraction, followed by hybrid brachytherapy using Venezia applicators. This technique, which involves the use of transvaginal needles in addition to tandem and ovoid, allows high doses (85–90 Gy) to be delivered to parametrial and surrounding involved pelvic tissue, which is otherwise not achievable with intracavitary brachytherapy alone.

The primary objective of this study was to report the severity of acute GI and GU toxicity observed post concurrent chemoradiotherapy (CCRT) and IGBT using a hybrid technique in patients with locally advanced cervical cancer, employing common terminology scoring criteria. The use of interstitial needles combined with tandem, and ovoid facilitates a more uniform dose distribution, particularly in cases with parametrial extension, without exceeding the tolerance limits of OARs. The secondary objective was to evaluate the efficacy of this approach in achieving a treatment response, thereby promoting a safer technique for patients. This is the first hybrid brachytherapy institution experienced in Pakistan.

## Materials and methods

A prospective analysis was conducted in the Radiation Oncology Department of Atomic Energy Cancer Hospital NORI Islamabad on 41 patients from June 2022 to January 2024 diagnosed with locally advanced squamous CA of the cervix with the Ethical Review Board approval reference number 2(72)/88/RTMC. All procedures were in accordance with institutional and national ethical standards and the 1964 Helsinki Declaration and its later amendments or comparable ethical standards.

**Figure 1.**
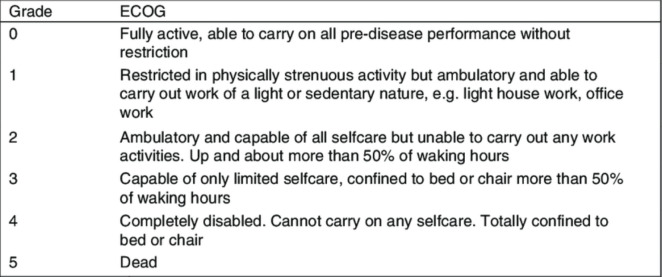
ECOG [25].

**Figure 2.**
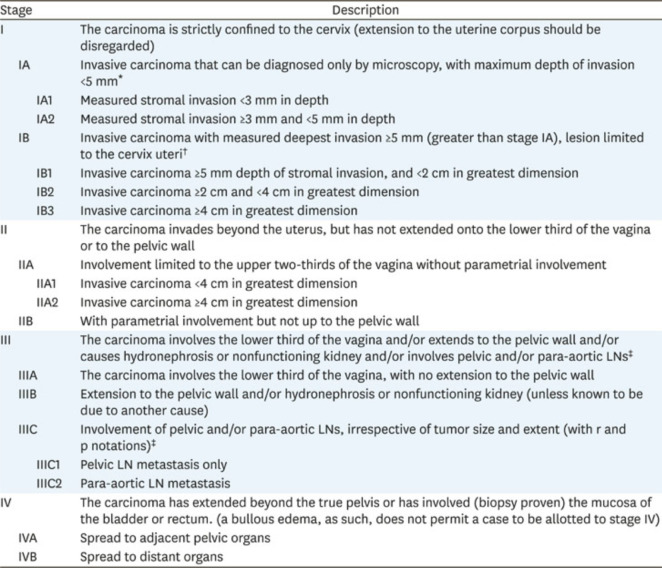
FIGO classification for cervical cancer [26].

After obtaining informed consent, patients were treated with external beam radiotherapy using the IMRT technique followed by brachytherapy. All patients aged >25 to <70 years, staged according to the 2018 International Federation of Gynecology and Obstetrics (FIGO) staging system, with an intact uterus and locally advanced cervical cancer (FIGO stage IB3/IIA2-IVA), a biopsy-proven histopathological diagnosis of squamous CA cervix, an Eastern Cooperative Oncology Group (ECOG) score of 1 (good performance status), and normal haematological and biochemical profiles were included (Figures 1 and 2). Those with early stage (microscopic cervical cancer) that is surgically operable or metastatic disease, previous pelvic radiotherapy, and comorbid conditions like uncontrolled hypertension, previous myocardial infarction restricting oncological intervention, co-existing second malignancy, and history of previous malignancy except for squamous CA and basal CA of the skin were excluded. All patients were tested for HPV at the start of the study as well as at 3 months follow-up using Pap smear.

A total of 140 fractions of hybrid brachytherapy using the Venezia applicator were performed on 41 patients. Each patient was treated using 2 interstitial implants and 3–4 fractions of brachytherapy post-EBRT (2 fractions were given using one implant). Patients were monitored for acute GI and GU side effects every 2 weeks during external beam radiotherapy and later at 6 weeks and 3 months post-brachytherapy, making a total of five visits per patient. Toxicity grades were assessed using Common Terminology Criteria for Adverse Events (CTCAE) Version 5.0. Response to treatment was defined using the Response Evaluation Criteria in Solid Tumours (RECISTs) Criteria 1.1 as stable disease, partial response, complete response and progressive disease at 3 months follow-up [10].

At presentation, a detailed history and physical examination were conducted followed by staging workup that included contrast-enhanced MRI of the pelvis and CT of the chest and abdomen. Baseline investigations such as blood complete profile, renal scan, and liver function tests were ordered. Proctoscopy and sigmoidoscopy were advised for patients whose MRI showed indistinct fat planes with the bladder and rectum. Patients with good performance status and normal blood profiles, fulfilling the inclusion criteria, were scheduled for CCRT followed by IGBT.

All patients received external beam radiotherapy with doses between 45 and 50.4 Gy at 1.8 Gy per fraction. Patients with an estimated glomerular filtration rate (eGFR) of more than 60 ml/min were also given 40 mg/m^2^ weekly cisplatin. Those with stage IIIC1 disease or higher received a simultaneous integrated boost to residual nodes. Patients were simulated using a comfortably full bladder and an empty rectum. Cone beam CT was done twice a week for treatment verification. IMRT technique was used for treatment planning to achieve tolerance doses for organs at risk (OARs). For hybrid brachytherapy, patients were admitted to the ward 48 hours before the procedure, with vitals recorded every 8 hours and a soft diet maintained. NPO was enforced 8–12 hours before the procedure, with Kleen enemas given twice before treatment. Haematological and biochemical profile was repeated weekly during EBRT, and patients were evaluated for acute GI and GU side effects every 2 weeks during treatment and at 6 weeks and 3 months follow-up.

After completing EBRT, an MRI of the pelvis was repeated to assess the response and plan further treatment. Clinical and radiological assessments post-EBRT determined suitability for brachytherapy, including total dose, number of fractions, dose per fraction, and the number of needles. Most patients were treated using hybrid Venezia applicators, except those with stage IIA or less, who were treated with only tandem and ovoids due to no parametrial involvement.

Epidural anesthesia was used for patient comfort and pain relief. Under aseptic conditions, patients were prepared, and the bladder was filled to a maximum of 15 cc after catheterisation. Hybrid brachytherapy applicators were inserted under real-time image guidance using an ultrasound scan by a consultant radiologist.

Treatment planning used MRI for better delineation of the tumour and OARs. High-risk clinical target volume (HR-CTV) and Internal radiation clinical target volume (IR-CTV) were contoured per Groupe Européen de Curiethérapie and the European Society for Radiotherapy & Oncology Gynecology group (GYN-GEC-ESTRO) guidelines [7]. OARs, such as the bladder, rectum, and sigmoid, were separately contoured. Response to treatment was observed radiologically using both MRI and clinically at the time of applicator insertion.

Brachytherapy plans aimed for a dose received by 90% of volume (D90) of more than 85 Gy to HR-CTV and 60 Gy to IR-CTV while keeping D2cc (minimum dose to the most irradiated 2 cc) as low as possible. Dose constraints as Equivalent Dose in 2 Gy per fraction (EQD2) for OARs were followed using EMBRACE and Retro-EMBRACE guidelines for the bladder (90 Gy) and rectum/sigmoid (70–75 Gy) [6, 11]. This dose was derived after adding the dose received by these structures during external beam radiotherapy.

Data were analysed using IBM-SPSS version 26. Descriptive statistics were used for patient characteristics. Frequency and percentages presented categorical variables. The severity of acute GI and GU side effects was reported using CTCAE Version 5.0 and described in tabular form. Fisher’s exact test was used to find the association between clinicopathological variables and acute side effects (*p*-value <0.05 was considered statistically significant).

## Results

All 41 patients were diagnosed with squamous CA of the cervix and had an ECOG score of 1 across all five visits. Nineteen patients (46.3%) received a total dose of 80–90 Gy, and 22 patients (53.7%) received more than 90 Gy to the HRCTV D90. Table 1 presents the characteristics of cervical CA patients across the five visits.

All patients underwent EBRT, with 4 (9.8%) receiving 50.4 Gy and 37 (90.2%) receiving 45 Gy, at a dose per fraction of 1.8 Gy. Concurrent chemotherapy was administered to 36 patients (87.8%), while 5 (12.2%) did not receive it. Among the 68.3% of HPV-positive patients, 46.3% converted to negative cases with complete response. No significant correlation was found between disease staging and treatment response (*p* = 0.36).

The total duration of radiotherapy treatment (EBRT and brachytherapy) was 6 weeks for 5 patients (12.2%), 7 weeks for 17 patients (41.5%), and 8 weeks for 19 patients (46.3%). All patients were treated with hybrid brachytherapy. Fifteen patients (36.6%) received 3 fractions of brachytherapy, and 26 patients (63.4%) received 4 fractions. Complete responses were observed in 12.1%, 29.2%, and 24.3% of patients after 6, 7, and 8 weeks of treatment, respectively, with partial responses in 0%, 21.7%, and 17% of patients. No significant relationship was found between the number of fractions of brachytherapy and treatment response.

**Figure S1.**
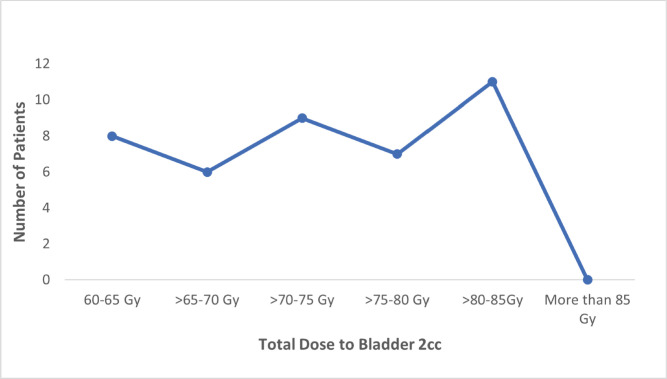
Number of patients receiving total dose (EQD2) to bladder 2 cc.

**Figure S2.**
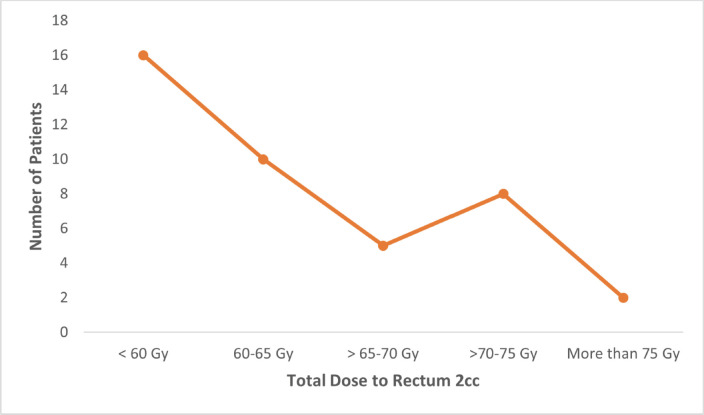
Number of patients receiving total dose (EQD2) to rectum 2 cc.

**Figure S3.**
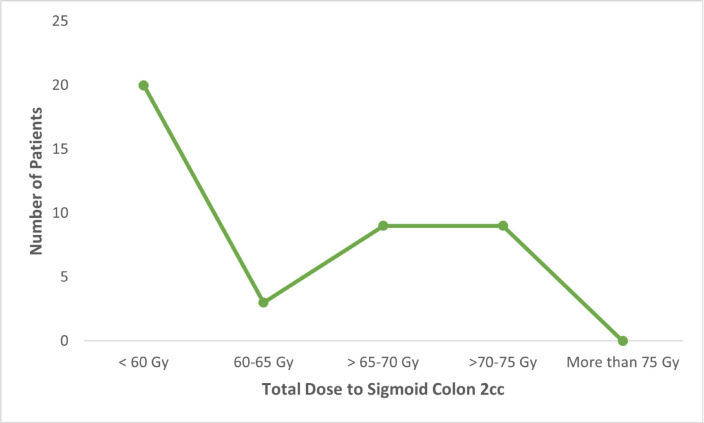
Number of patients receiving total dose (EQD2) to sigmoid colon 2 cc.

Fisher’s Exact test showed no significant relationship between age and final treatment response (*p* = 0.73) or between EBRT dosage and final response (*p* = 0.65). However, the area of residence significantly correlated with treatment response (*p* = 0.001). Figures S1–S3 show the total EQD2 given to the bladder, rectum, and sigmoid. Middle-class patients exhibited higher rates of complete (56%) and partial (17%) responses compared to lower and upper-class patients, though the Fisher exact test indicated an insignificant association (*p* = 0.158).

By the fifth visit, 56.1% of complete responders and 29.2% of partial responders experienced Grade 0 toxicity for blood in urine, blood in stool, nausea, and vomiting. For painful urination, 53.6% of complete responders and 19.5% of partial responders had Grade 0 toxicity, while 12.2% had Grade 1. Frequency of urination toxicity was Grade 0 in 26% of complete responders and 8% of partial responders, with 12.1% showing Grade 1. Of the 27 patients with complete responses, 20 had Grade 0 abdominal pain and 7 had Grade 1, while 10 partial responders had Grade 0 abdominal pain. Additionally, 58.5% of patients with complete responses exhibited Grade 0 stool frequency, while 7.3% had Grade 1. Table 2 shows a decrease in side effects observed in cervical CA patients after brachytherapy from the first to the last visit. At the study’s conclusion, complete response was observed in 27 patients (65.9%), partial response in 12 patients (29.3%), and stable disease in 2 patients (4.9%), according to RECISTs Criteria 1.1 [10].

## Discussion

This study utilised image guided hybrid brachytherapy (first in our country) to explore the acute toxicity profile and the response of patients with locally advanced squamous CA of the cervix. Hybrid brachytherapy, combining interstitial implants with tandem and ovoid (IC/IS), allows higher doses (85–90 Gy) to be delivered to the gross disease without increasing OAR tolerance doses, improving dose coverage of the HR-CTV. Our study showed that we were able to achieve doses in the order of 80–90 and >90 Gy to HRCTV without exceeding the tolerance doses to OARs. Consequently, this intervention adheres to a higher efficacy and safety profile.

Radiotherapy is the primary modality for treating squamous CA of the cervix, which combines EBRT and brachytherapy to deliver high doses to the tumour while sparing organs at risk. Brachytherapy is integral to cervical cancer management, improving local control (LC) and OS [12]. Modern radiotherapy techniques like IMRT, hybrid applicators and plastic catheters have revolutionised treatment, allowing higher, more conformal dose delivery with fewer side effects. The RetroEMBRACE study also showed that hybrid brachytherapy increased 3-year LC by 10% without increasing late toxicities compared to intracavitary brachytherapy [11].

The most common stage at presentation in our study was IIIC1 (39%), followed by stage IIB (29.3%). In a study from AKUH in Pakistan, the most common stage was IIB [13] showing that most patients in our country present with locally advanced disease stage. As per previous literature, the stage of disease presentation impacts treatment-related toxicity as increased tumour size results in greater toxicity and less response to treatment [14], but no statistically significant association was found between disease stage and toxicity in our study. Similar results were reported by Chi *et al* [15] from China but this needs further validation. Previous studies have indicated that social status significantly affects treatment response and overall outcomes in cervical cancer patients, with higher social class women more likely to participate in regular screening and have better healthcare access, leading to early detection and better treatment [16]. Our analysis revealed that middle-class patients had higher rates of complete (47.8%) and partial (13%) responses compared to lower and upper-class patients, with statistically insignificant association. However, residents of Rawalpindi and Islamabad showed higher rates of complete response as per the RECIST criteria, but no comparative studies are available in the literature. Response evaluation using RECIST Criteria is an important and more means of evaluating treatment response and predicting clinical outcomes as reported in the previous literature [17] it is comparable to the standard clinic-radiological evaluation. Moreover because of the high diagnostic yield of MRI patient are spared from undergoing unnecessary biopsies.

In our study, out of the 41 patients, almost half of the patients received doses above 90 Gy. We were able to achieve complete response in 27 patients at the 3-month follow-up. The EMBRACE protocol recommends a D90 to the HRCTV of 87 Gy or higher for effective LC (above 95%) [6]. Since all patients received doses above 80 Gy, no dose-response relationship was found. Although LC depends on disease stage, our results showed no significant relation, like the RetroEMBRACE study [11].

An interesting finding was that 16 out of 23 HPV-positive patients on pre-treatment Pap smears became negative post-treatment, which may have clinical implications and affect prognosis, although no relevant data is available.

Previous studies have shown a significant association between treatment duration and response in patients treated with chemoradiotherapy, ideally treatment should be completed within 7–8 weeks [12]. In our study, 10 patients (43.5%) completed treatment within 8 weeks, 11 patients (47.8%) in 7 weeks, and only 2 patients in 6 weeks, with no significant association found between treatment duration and response. Studies have shown that patients with higher baseline haemoglobin levels have better treatment outcomes and higher tumour control rates, as adequate oxygenation is crucial for effective radiotherapy, enhancing radiation-induced cell death [18]. However, our results were not consistent with previously published literature.

The most common acute GI and GU toxicities were Grade 1. No Grade III or IV toxicities were observed. None of the patients experienced blood in urine or stool. A study from Singapore by Sommat *et al* [18] reported that only one in 33 patients developed Grade 3 cystitis, with no Grade 4 toxicities observed in patients treated with MRI-based hybrid brachytherapy. Prior to the use of 3D conformal radiotherapy and IGBT, most patients receiving radiotherapy for squamous CA cervix experienced much greater toxicities and most of them had high-grade toxicities that required prolonged hospital stays and frequent treatment interruptions. The use of newer radiotherapy techniques such as IMRT, and hybrid brachytherapy (using interstitial implants) has resulted in better treatment outcomes such as improved locoregional control, it also allows simultaneous delivery of boost dose to sites such as involved nodes with a much-improved toxicity profile, reducing both acute and late side-effects [19, 20]. Pelvic radiotherapy mainly affects GI and GU system followed by haematological system [8, 20]. Our study mainly focused on the acute GU and GI toxicity. Most of our patients experienced mild (Grade 1 and 2) toxicities that did not require any hospital admission or treatment gaps and were easily managed with supportive care.

The advantage of using Venezia applicators for hybrid brachytherapy, is eased dose delivery and allowed better reshaping of isodose curves resulting in more conformal dose distribution. Previous studies have shown that hybrid brachytherapy achieves better dosimetric parameters than intracavitary brachytherapy alone, resulting in better tumour control without increasing OAR tolerance limits [13]. The Vienna group first described the dosimetric advantage of combined IC/IS applicators, recommending their use for tumours with parametrial invasion, suboptimal response following chemoradiation, and unfavourable topography [21, 22]. Harkenrider *et al* [23] demonstrated that using hybrid applicators, the median HR-CTV D90 increased from 88 to 92.9 Gy, while the median D2cc to rectum significantly decreased from 69.3 to 62.6 Gy (*p* = 0.01), with a nonsignificant decrease in bladder D2cc. Another study using the Venezia hybrid applicator found significant dose differences between hybrid and IC applicators for HR-CTV D90, bladder, and rectal D2cc, but not for the sigmoid [24] resulting in a good toxicity profile. Similarly in our study, the doses to OARs were well within the tolerance limits with the mean dose to 2 cc of rectum and sigmoid colon being less than 60 Gy and to bladder 80–85 Gy as mentioned in Table 3. Venezia applicators are particularly effective in treating advanced-stage disease even Stage IVA (involving the bladder and rectal mucosa). The ease of use and handling of interstitial needles allows accurate placement inside the tumour and the highly conformal distribution of isodose curves results in minimal doses to surrounding OARs [24].

This study was conducted to augment the knowledge of hybrid brachytherapy and suggest its utility as the primary method for treating locally advanced cervical cancer. The improved efficacy, cost-effectiveness and toxicity profile of image-guided hybrid brachytherapy will be particularly helpful for the middle and low-income countries with high disease burden and limited resources.

## Limitations

This study faced challenges, including a small sample size, as many patients did not follow up until 3 months, being referred from other centres and completed follow-up at primary care hospitals. Additionally, a fixed brachytherapy regimen was not used, with different fractionation schedules and doses per fraction employed to achieve D90 above 85 Gy. Random sampling was also not performed.

## Conclusion

Hybrid brachytherapy, using both intracavitary and interstitial techniques with Venezia applicators, offers significant benefits for the treatment of cervical cancer, particularly in low- and middle-income countries like Pakistan, where the incidence of cervical cancer is increasing, and many patients present with advanced stages of the disease.

1. Challenges with intracavitary brachytherapy alone:
  - Intracavitary brachytherapy alone has been associated with suboptimal disease control in patients with locally advanced cervical cancer.
2. Advantages of hybrid brachytherapy
  - Improved toxicity profile: Hybrid brachytherapy demonstrates a better toxicity profile compared to intracavitary brachytherapy alone.
  - Enhanced treatment outcomes: It leads to better treatment outcomes and disease control.
3. Use of RECIST criteria
  - RECIST provides a standardised, non-invasive method for assessing the response to treatment, facilitating consistent and objective evaluations.
4. Cost-effectiveness of Venezia applicators
  - Venezia applicators are cost-effective and offer excellent disease control without increasing morbidity.

## List of abbreviations

CTCAEs, Common terminology criteria for adverse events; EBRT, External beam radiation therapy; ECOG, Eastern Cooperative Oncology Group; GI, Gastrointestinal; GU, Genitourinary; IGBT, Image guided brachytherapy; IMRT, Intensity modulated radiation therapy; HRCTV, High-risk clinical target volume; RECISTs, Response evaluation criteria in solid tumours.

## Conflicts of interest

The authors declared no potential conflicts of interest with respect to the research, authorship, and/or publication of this article.

## Funding

The authors received no financial support for the research, authorship, and/or publication of this article.

## Consent for publication

Consent for publication without any identifying details was obtained from the participants.

## Ethical approval

The Research Training and Monitoring Cell (RTMC) at Atomic Energy Cancer Hospital (AECH) NORI granted ethical approval before the study was conducted with reference number 2(72)/88/RTMC. Since there was no requirement for a human biological sample, informed consent was obtained from the patients by the interviewers before participation, following ethical rules for epidemiological research in Japan, rather than obtaining written consent. Furthermore, patient autonomy and confidentiality were maintained in accordance with fundamental Law 41/2002, which governs patient autonomy and health documentation and information-related rights and obligations.

## Availability of data and material

Data are available for all 41 participants of this study.

## Figures and Tables

**Table 1.** Characteristics of cervical CA patients.

Characteristics	Number (*n*)	Percentage (%)
Age > 25 to ≤ 45 >45 to ≤ 70	11 30	26.8% 73.2%
Socio-economic status Lower class Middle class Upper class	07 32 02	17.1% 78% 4.9%
Residence Islamabad/Rawalpindi KPK (Khyber Pakhtunkhwa) Upper Punjab Lower Punjab Gilgit/Baltistan Kashmir	22 01 11 02 0 05	53.7% 2.4% 26.8% 4.9% 0% 12.2%
HPV 16 pre-treatment status Positive Negative Not done HPV 16 post-treatment status Positive Negative Not done	28 09 04 09 28 04	68.3% 22% 9.8% 22% 68.3% 9.8%
FIGO classification of cervical CA IB3 IIA2 IIB IIIA IIIB IIIC1 IIIC2 IVA	0 5 12 4 0 16 0 4	0% 12.2% 29.3% 9.8% 0% 39.02% 0% 8.7%
Hb level < 12 g/dL >12 to <14 g/dL	20 21	48.8% 51.2%
Platelets count <150,000/mcL 150,000 to 300,000/mcL >300,000 to 450,000/mcL	02 32 07	4.9% 78% 17.1%
Absolute neutrophil count <1,500 >1,500	02 39	4.9% 95.1%
RFTs (Renal function tests) creatinine: <1 mg/dL >1 mg/dL	36 05	87.8% 12.2%
eGFR <60 mL/min 60-90 mL/min >90 mL/min	05 18 18	12.2% 43.9% 43.9%

**Table 2.** Side effects of EBRT and brachytherapy with each visit.

Side effects	Number of participants (%) at:				
	Visit 1	Visit 2	Visit 3	Visit 4	Visit 5
Nausea Grade 0: Grade 1: Grade 2:	23 (56.1%) 17 (41.5%) 1 (2.4%)	19 (46.3%) 20 (48.8%) 2 (4.9%)	22 (53.7%) 17 (41.5%) 2 (4.9%)	37 (90.2%) 4 (9.8%) 0	37 (90.2%) 4 (9.8%) 0
Vomiting Grade 0: Grade 1: Grade 2:	33 (80.5%) 7 (17.1%) 1 (2.4%)	35 (85.4%) 4 (9.8%) 2 (4.9%)	34 (82.9%) 1 (2.4%) 6 (14.6%)	39 (95.1%) 0 2 (4.9%)	37 (90.2%) 4 (9.8%) 0
Abdominal pain Grade 0: Grade 1: Grade 2:	32 (78%) 9 (22%) 0	20 (48.8%) 18 (43.9%) 3 (7.3%)	17 (41.5%) 16 (39%) 8 (19.5%)	25 (61%) 14 (34.1%) 2 (4.9%)	32 (78%) 7 (17.1%) 2 (4.9%)
Frequency of stool Grade 0: Grade 1: Grade 2:	33 (80.5%) 5 (12.2%) 3 (7.3%)	23 (56.1%) 12 (29.3%) 6 (14.6%)	22 (53.7%) 14 (34.1%) 5 (12.2%)	36 (87.8%) 5 (12.2%) 0	38 (92.7%) 3 (7.3%) 0
Blood in stool Grade 0: Grade 1:	40 (97.6%) 1 (2.4%)	41 (100%) 0	40 (97.6%) 1 (2.4%)	41 (100%) 0	41 (100%) 0
Painful urination Grade 0: Grade 1: Grade 2:	25 (61%) 16 (39%) 0	18 (43.9%) 19 (46.3%) 4 (9.8%)	13 (31.7%) 16 (39%) 12 (39.3%)	16 (39%) 23 (56.1%) 2 (4.9%)	32 (78%) 7 (17.1%) 2 (4.9%)
Frequency of urination Grade 0: Grade 1: Grade 2:	32 (78%) 9 (22%) 0	28 (68.3%) 8 (19.5%) 5 (12.2%)	21 (51.2%) 12 (29.3%) 8 (19.5%)	28 (68.3%) 11 (26.8%) 2 (4.9%)	36 (87.8%) 5 (12.2%) 0
Blood in urine Grade 0:	43 (100%)	43 (100%)	43 (100%)	43 (100%)	43 (100%)

**Table 3.** Dosimetric parameters of patients treated with EBRT + brachytherapy.

Mean EBRT dose	50.4 Gy (9.8%)	45 Gy (90.2%)	-
Dose to D90 HRCTV	80–90 Gy (46.3%)	>90 Gy (53.7%)	-
Number of fractions of brachytherapy	3 fractions (36.6%)	4 fractions (63.4%)	-
Total treatment time (EBRT+Brachytherapy)	6 weeks (12.2%)	7 weeks (41.5%)	8 weeks (46.3%)
Mean dose to bladder	80–85 GY		
Mean dose to rectum	< 60 Gy		
Mean dose to sigmoid	< 60 Gy
